# Comparison of Gross Lesions in Poultry Naturally Infected With High Pathogenicity Avian Influenza H5N6 and H5N1 Viruses in South Korea, 2023–2025

**DOI:** 10.1155/tbed/1736453

**Published:** 2025-11-25

**Authors:** Hye-Ryoung Kim, Kwang-Nyeong Lee, Youn-Jeong Lee, Moon Her

**Affiliations:** ^1^Avian Disease Division, Animal and Plant Quarantine Agency, 177 Hyeoksin 8-ro, Gimcheon-si, Gyeongsangbuk-do, Republic of Korea; ^2^Avian Influenza Research and Diagnostic Division, Animal and Plant Quarantine Agency, 177 Hyeoksin 8-ro, Gimcheon-si, Gyeongsangbuk-do, Republic of Korea

**Keywords:** chickens, ducks, gross lesion, high pathogenicity avian influenza, pathology

## Abstract

High pathogenicity avian influenza (HPAI) is an acute infectious disease of poultry and wild birds that has been occurring worldwide and has been controlled in many countries by culling birds on farms with disease outbreaks. We compared the gross lesions observed in influenza A (H5Nx) virus-positive cases of chickens and ducks in South Korea between 2023 and 2025. A total of 49 outbreaks were identified, comprising 34 cases in chickens and 15 cases in ducks, with both H5N1 and H5N6 subtypes detected. The lesions observed most frequently in chickens included splenomegaly and splenic necrosis, followed by tracheal congestion and pancreatic necrosis. In ducks, tracheal congestion was the most common lesion, followed by pancreatic necrosis and splenomegaly; hemorrhage and/or necrosis were also observed in the liver, ovarian follicles, heart, and lungs. Gross lesions in poultry caused by the H5N1 virus during the 2024–2025 season were observed more frequently than those associated with H5N6 virus in 2023. HPAI cases were characterized by ≥2 HPAI-typical lesions, such as pancreatic necrosis, splenic necrosis, and ovarian follicular hemorrhage, or the presence of HPAI–associated lesions in ≥3 different organs, even in the absence of HPAI-typical lesions. Assessing gross lesions in HPAI cases is crucial for guiding immediate disease control measures, including imposing movement restrictions on suspected farms, while awaiting confirmation by genetic testing and sequencing.

## 1. Introduction

High pathogenicity avian influenza (HPAI) has spread worldwide since 2020, causing numerous epizootic events in poultry across Asia, Europe, and North and South America. Clade 2.3.4.4 H5 HPAI virus (HPAIV) has predominated globally since 2014, with the 2.3.4.4b virus causing HPAI outbreaks in wild birds and poultry including South Korea since 2020 [1–3]. Within this subclade of H5Nx avian influenza virus (AIV; Orthomyxoviridae, *Alphainfluenzavirus influenzae*), a wide range of host species, including poultry, wild birds, mammals, and even humans have been affected, leading to significant economic losses in the poultry industry and raising serious public health concerns [4–6].

In South Korea, HPAI reemerged during the 2024–2025 winter season following outbreaks in the 2023–2024 winter season, caused by H5N1 and H5N6 viruses of the 2.3.4.4b clade. The first suspected HPAI case was reported on December 3, 2023, at a meat duck farm, where H5N6 and H5N1 HPAIV were confirmed simultaneously [7]. By December 25, 2023, 16 HPAI-positive cases (15 H5N6 and 1 H5N6/N1) had been identified in commercial farms. In 2024, the index case of HPAI was diagnosed at a layer farm on October 29, and by January 30, 2025, 33 H5N1 HPAI-positive cases had been confirmed in commercial farms. Most of the cases analyzed in our study were layer and meat ducks, with a small number involving broiler-breeder, duck breeder, and Korean native chickens raised for both meat and egg production (Supporting Information 1: Table S1 and Supporting Information 2: Table S2). However, the present study did not include all of the HPAI outbreaks from each season. Cases detected solely through routine surveillance or pre-slaughterhouse testing, or those with an epidemiologic link but lacking clinical signs or official notification, were excluded.

Here, we investigated whether the organ-specific macroscopic lesions in HPAI outbreaks during 2023–2025 in South Korea differed by host species (chickens vs. ducks) or by virus subtypes (H5N1 vs. H5N6).

## 2. Materials and Method

In most cases, suspected HPAI cases were reported by farm owners to their local county offices based on increased mortality, decreased egg production, and reduced feed intake. Necropsy of deceased birds is a crucial step in the preliminary assessment of HPAI through gross lesion examination and serves as a valuable tool for animal health surveillance [8, 9]. Five dead birds per case were collected by veterinary inspectors dispatched by local governments and submitted to the regional Animal Disease Control (ADC) office with jurisdiction over the affected farm. Necropsies were conducted at biosafety Level 3 facilities. Gross lesions in deceased birds were jointly evaluated by the local ADC and the Animal and Plant Quarantine Agency (APQA; Gimcheon-si, Republic of Korea) using a standardized and systematic approach, with photographs taken during the necropsies shared digitally for verification; the description of gross lesions was confirmed by a specialist from the APQA to ensure consistency. Final diagnoses were confirmed using subtype-specific real-time polymerase chain reaction (PCR) and hemagglutinin (HA) cleavage site sequencing [10]. In cases confirmed as HPAIV, disease control measures, such as culling and movement restrictions, were promptly implemented. If HPAIV was not confirmed, the local ADC performed further testing for other pathogens, adhering to diagnostic protocols [11]. After necropsy, all remaining carcasses and biological materials were disposed of by incineration at the local ADC laboratories that conducted the necropsies or through certified medical waste disposal companies authorized for biohazard waste treatment. Ethical approval was not required for this study because no live animals were used. All carcasses were collected postmortem as part of official diagnostic and surveillance activities conducted by the APQA and regional ADC offices in South Korea, in accordance with national biosafety and animal health regulations.

A total of 49 poultry farms confirmed with HPAI were evaluated for gross lesions in chickens and ducks during the 2023 and 2024–2025 outbreak seasons. Among chickens, the majority of cases were reported in layer farms (10 farms in 2023 and 17 farms in 2024–2025), followed by broiler-breeder (two farms in 2023 and two farms in 2024–2025) and layer-breeder (one farm in 2024–2025). In ducks, the majority of cases were reported in meat-type farms (three in 2023 to nine in 2024–2025), followed by breeder duck farms (one in 2023 to two in 2024–2025). Overall, 16 farms were affected in 2023, while 33 farms were affected in the 2024–2025 season (Supporting Information 1: Table S1 and Supporting Information 2: Table S2).

Gross changes that would be suggestive of HPAI included the following: spleen: enlargement and multifocal to coalescing pale necrotic foci; pancreas: multifocal white necrotic foci and congestion; liver: friability, hemorrhage, and scattered pale necrotic foci; trachea: congestion and hemorrhage; ovarian follicle: hemorrhage and rupture; heart: petechiae on the epicardium, myocardium, pericardiac fat, and pallor of myocardium; lung: congestion; other organs: petechiae in their serosae, petechiae in the celomic fat, ascites, hemorrhage in the proventricular mucosa, and hemorrhage in the mucosa of the cecal tonsil.

## 3. Results

**Figure 1:**
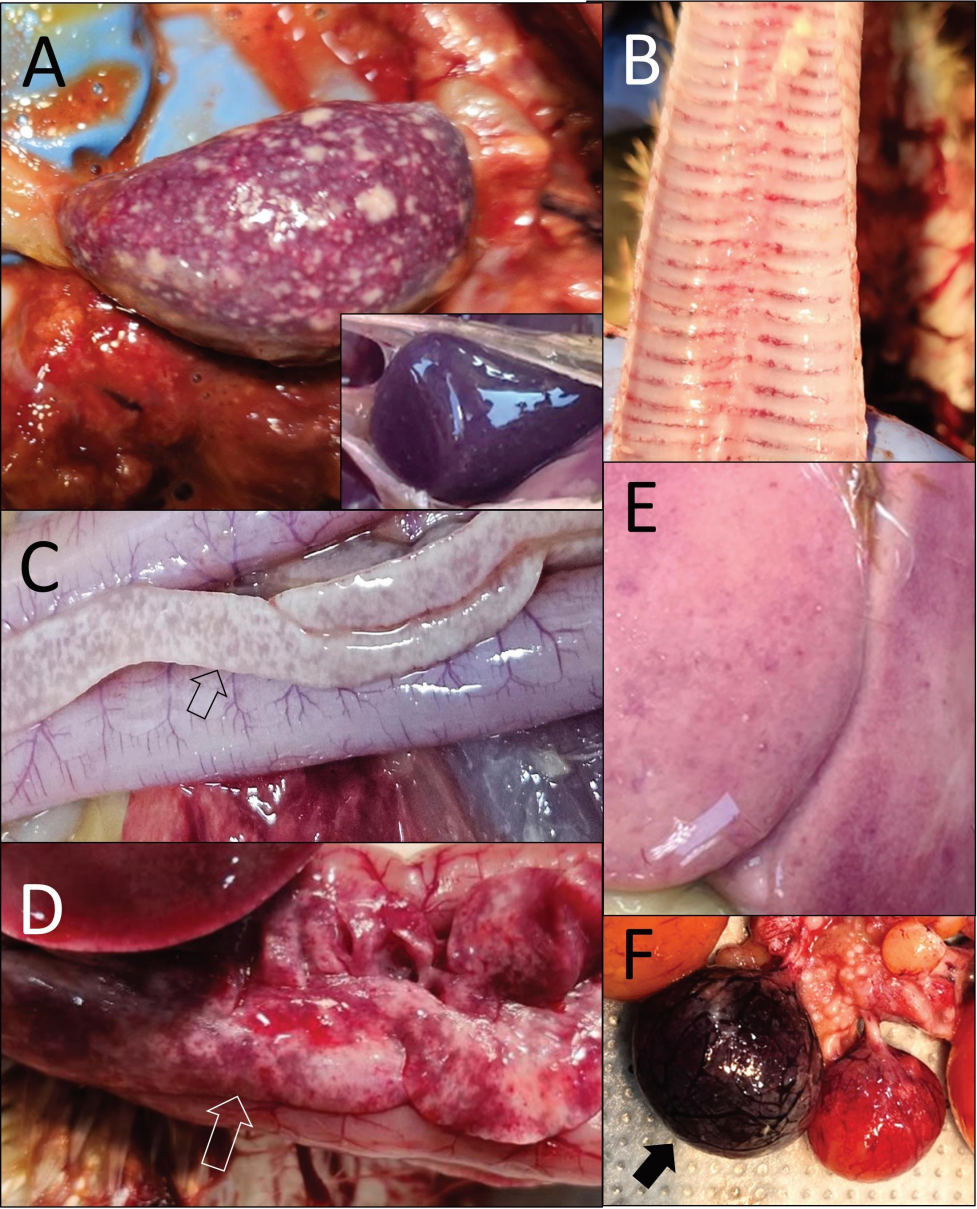
Gross lesions in poultry infected with the H5N1 high pathogenicity avian influenza virus during the 2024–2025 winter season in South Korea. (A) Multifocal to coalescing splenic necrosis in a chicken. Inset: miliary splenic necrosis in a duck. (B) Tracheal hemorrhage in a duck. (C) Pancreatic necrosis (arrow) in a chicken. (D) Pancreatic necrosis with hemorrhage (arrow) in a duck. (E) Swollen and pale liver of a chicken with miliary necrohemorrhagic foci. (F) Ovarian follicular hemorrhage (arrow) in a chicken.

**Figure 2:**
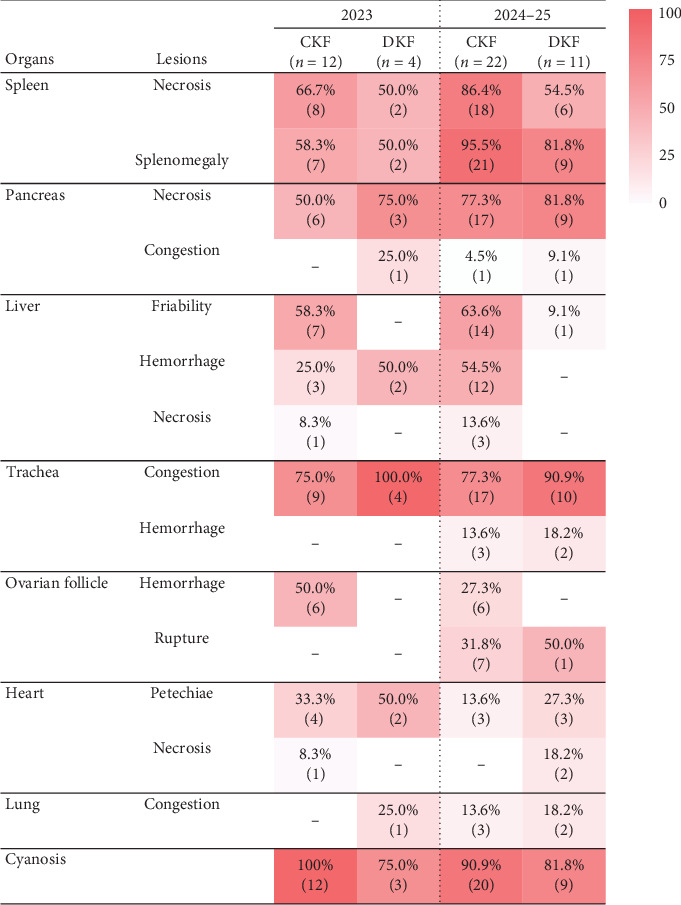
Incidence of gross lesion in HPAI-positive poultry farm cases (2023–2025). CKF, chicken farm; DKF, duck farm.

The most frequently observed lesion suggestive of HPAIV infection in chickens and ducks between 2023 and 2025 was splenic necrosis, characterized by fine or pale necrotic foci and mottled parenchyma. In chickens during the 2024–2025 season, splenic necrosis was particularly severe, with high- density and irregularly sized necrotic foci (Figure 1A). When comparing the 2023 and 2024–2025 seasons, the detection rate of splenic necrosis in HPAI-positive chicken farms increased markedly from 66.7% to 86.4%, while that in duck farms showed a slight increase in the 2024–2025 season. Splenomegaly was also frequently observed in HPAIV-infected poultry; 95.5% chicken farms and 81.8% duck farms had this lesion during the 2024–2025 season, compared to 58.3% of chicken farms and 50.0% of duck farms that tested positive for HPAI in 2023 (Figure 2).

Gross lesions in the trachea of HPAI-infected birds were edema, congestion, or hemorrhage of the intercartilaginous mucosa (Figure 1B). Tracheal congestion was consistently observed, with slightly higher frequencies in ducks (100% and 90.9%) compared with chickens (75.0% and 77.3%) between the seasons of 2023 and 2024–2025 (Figure 2).

Another key lesion was pancreatic necrosis, grossly seen as focal hemorrhagic or whitish-gray necrosis within the pancreatic parenchyma (Figures 1C,D). This lesion was found in both species, with a slightly higher prevalence in ducks (81.8%) than in chickens (77.3%) during 2024–2025, and similar trends were noted in 2023. The detection rate in the 2024–2025 season was slightly higher than in 2023.

Liver lesions in HPAIV-infected chickens included hepatomegaly and friability, often leading to rupture with coelomic hemorrhages and blood clots, as well as petechial hemorrhages within parenchyma. Hepatic necrosis, characterized by white or pale yellow spots, was almost always accompanied by hepatomegaly (Figure 1E). In 2024–2025, the detection rate for hepatic hemorrhage and hepatic necrosis in chickens doubled compared to 2023, at 54.5% and 3.6%, respectively. No hepatic necrosis was observed in ducks, and hepatic hemorrhage was only seen in 50% of duck farms in the 2023 season (Figure 2).

In layers and breeder (chickens and ducks), ovarian follicle lesions included intrafollicular hemorrhages filled with yolk and blood and ruptured follicles appearing flaccid and wrinkled with yolk leakage into the coelom (Figure 1F). Follicular hemorrhage was observed only in chickens (up to 50% of cases), while follicular rupture was observed in both chickens (31.8%) and ducks (50%) in the 2024–2025 season.

Petechial epicardial hemorrhage were more frequently detected in the 2023 season than in 2024–2025. Myocardial necrosis was found in one chicken case in 2023 and two cases of ducks in 2024–2025. Pulmonary congestion was relatively rare, while tracheal congestion was frequently observed in both species.

Cyanosis was observed in most confirmed HPAI cases, manifesting in the comb in chickens and the beak in ducks, with little difference in the frequency between species or across seasons.

## 4. Discussion

The findings of this study highlight distinct gross lesion patterns in dead chickens and ducks infected with HPAIV in South Korea between 2023 and 2025. While splenic necrosis was the most consistent and useful lesion for suspecting HPAI, its prevalence and severity varied by season and species. Notably, the higher frequency and severity of splenic necrosis in chickens during the 2024–2025 season contrasts with reports from the United Kingdom in 2020–2021, where splenic necrosis was observed in <40 % of chickens and was absent in ducks infected with clade 2.3.4.4b HPAIV [8]. Splenomegaly, although common, is a nonspecific change that may result from other viral or bacterial infections, antigenic stimulation, or age-related factors [12, 13]. Without HPAIV negative control birds, interpreting the clinical significance of splenomegaly specifically in the context of HPAI is difficult.

Pancreatic necrosis in Galliformes was reported as a prominent lesion during the 2020–2021 HPAI epizootic in the UnitedKingdom and less frequently observed in Anseriformes infected with the 2.3.4.4b virus during the same outbreak [8]. However, in contrast to the UK cases, our findings indicate a notable increase in the detection of pancreatic necrosis in ducks during the 2024–2025 season. Pancreatic lesions can be caused by other viral diseases, such as adenovirus infection and Newcastle disease in chickens and turkeys but these etiologies were excluded in the present study based on clinical signs and molecular testing. Notably, these were observed in breeder ducks infected with HPAIV clade 2.4.4.4 in the UnitedKingdom in 2014, in meat-type and breeder ducks infected with HPAIV clade 2.3.2 in South Korea in 2010–2011, and in Baikal teals infected with HPAIV clade 2.3.4.4e in 2014 in South Korea. Such variation in incidence between seasons or host species may reflect differences in viral strain virulence, host susceptibility, and age or production type. These factors may lead to differences in the viral cell tropism, inflammatory response, or tissue damage of the HPAIV. Taken together, pancreatic necrosis can be considered a typical lesion in ducks indicative of HPAIV infection, irrespective of the virus strain [14–19].

Tracheal lesions, including hemorrhage and congestion, are not considered HPAI-typical lesions in chickens, as such changes can be caused by other respiratory viruses, including infectious bronchitis virus and low-pathogenic AIV (H9 subtype). Furthermore, similar lesions can also result from exposure to inhaled irritants, such as ammonia or hydrogen sulfide [20, 21]. Hepatic hemorrhage and friability in layers and breeders should be differentiated from fatty liver syndrome through histologic examination [22]. In the case of fatty liver, yellow-tan discoloration may be observed, but in some cases, only hepatic hemorrhage and friability are observed without discoloration, making gross differentiation difficult. Hemorrhagic, pale and friable livers in young broilers should also be differentiated from inclusion body hepatitis through PCR testing and histopathological examination [23]. In addition, natural infection with HPAIV can also lead to hepatic congestion, hemorrhage, and friability due to systemic viral replication and vascular damage. As such, gross hepatic lesions observed in the field should be interpreted with caution and confirmation through histopathology or molecular testing is essential to differentiate between fatty liver syndrome, HPAIV infection, and other hepatic disorders.

Follicular hemorrhage is not specific to HPAI and may also occur with systemic diseases, such as Newcastle disease or septicemia, and follicular rupture can be seen with low pathogenic AI, septicemic colibacilosis, fowl cholera, or systemic viral infections by infectious bronchitis virus. However, natural HPAI can directly involve the reproductive tract [24]. Experimental infection of adult hens with HPAIV produced early ovarian and oviduct lesions, follicular hemorrhage, and rupture within 36–72 h postinoculation, with viral antigen detected in granulosa and oviductal epithelium. The likely mechanism is viremia and local viral replication with endothelial injury, consistent with the pantropic and endotheliotropic behavior described for contemporary H5Nx clade 2.3.4.4 viruses [25, 26]. Therefore, gross reproductive tract lesions such as follicular hemorrhage and rupture may serve as early indicators suggestive of HPAI.

Petechial hemorrhages in the epicardium should be differentiated from bacterial infections such as *Riemerella* spp., *Erysipelothrix* spp., S*almonella* spp., and *Pasteurella multacida*. These bacterial infections typically present with additional gross lesions such as fibrinous pericarditis, perihepatitis, peritonitis, or polyserositis, which were not observed in the present HPAI cases. Myocardial discoloration and pulmonary congestion should be distinguished from postmortem changes [27, 28]. In addition, certain lesions, including epicardial hemorrhage and myocardial necrosis, appeared to show seasonal differences. The reason for this seasonal variation remains unclear, but several hypotheses such as differences in circulating HPAIV strains, variation in host immunity, concurrent environmental stressors, or coinfections with bacterial pathogens may be considered. Cyanosis of the combs and wattles in chickens is commonly associated with suspected HPAI cases [29], but it should be differentiated from postmortem change and protozoal infection causing anemia, such as *Histomonas meleagridis*, *Plasmodium* spp., and *Leucocytozoon* spp. Mucosa hemorrhages in the cecal tonsils and the proventriculus–gizzard junction were also observed in HPAI-positive cases. However, these lesions are not pathognomonic and can also occur in other viral diseases such as infectious bursal disease (typically in young birds with bursal hemorrhages), chicken infectious anemia (in younger flocks showing thymic atrophy and bone marrow aplasia), and Newcastle disease (often accompanied by hemorrhages in the proventriculus, cecal tonsils, and conjunctiva). In addition, septicemic bacterial infections, including those caused by *Escherichia coli*, *Salmonella* spp., and *Erysipelothrix* spp., may induce similar GALT hemorrhages. In such cases, concurrent gross findings such as fibrinous pericarditis, perihepatitis, or splenomegaly are more indicative of bacterial septicemia rather than HPAI.

Our results demonstrate that while both chickens and ducks exhibit a repertoire of common HPAI–associated lesions, the composition and relative prevalence of these lesions varied between species and outbreaks periods, with the most significant factor influencing these differences likely being the viral strains. Lesions in visceral organs varied by virus strain but were most consistently characterized by hemorrhages on serosal or mucosal surfaces and foci of necrosis within the parenchyma of visceral organs [29, 30]. Notably, pancreatic necrosis, splenic necrosis, and ovarian follicular hemorrhage were identified as HPAI-typical lesions and could serve as key diagnostic indicators for HPAI infection during necropsy. However, these HPAI-typical lesions were not always present in all HPAI-positive cases, as their manifestation may depend on host-related factors (age, immune status, and genetic background), environmental conditions (concurrent infections, management stress, or temperature), duration of infection, and viral virulence. For instance, younger or immunocompromised birds may show more severe and widespread hemorrhagic lesions, whereas well-conditioned flocks may present only mild or atypical signs [29, 31]. Similarly, environmental stressors or coinfections can exacerbate gross pathology, influencing lesion distribution and severity [29, 31]. A preliminary diagnosis of HPAI was made in 49 cases when two or more macroscopic HPAI-typical lesions and three or more other HPAI–associated lesions were observed. All cases satisfying these criteria were subsequently confirmed as HPAIV-positive through molecular diagnosis. The lesions designated here as “HPAI-typical” may be more accurately described as the most frequently encountered lesions during recent clade 2.3.4.4 outbreaks, providing practical diagnostic value while acknowledging their nonspecific nature.

Despite the HPAI viruses from the 2023–2025 South Korean outbreaks belonging to the same 2.3.4.4b clade with similar HA genes, we observed differences in the pathological lesions across the two seasons. This suggests that variations in pathogenesis or tissue tropism, possibly due to differences in internal genes (other than HA) or viral recombination, likely contributed to these discrepancies. Further genetic analyses and animal experiments are, therefore, essential to fully investigate the differences in pathogenicity between these two viral strains.

Although histopathology, immunohistochemistry, and in situ hybridization would provide valuable information to confirm viral tropism and differentiate coinfections, the present study was limited to gross pathological findings in cases confirmed as HPAI by virus isolation, identification, and sequencing. Nevertheless, we emphasize that documenting gross lesions remains important to raise suspicion of HPAI in the field and to enable implementation of immediate control measures while awaiting laboratory confirmation.

## 5. Conclusions

This study analyzed data on gross lesions from visceral organs in HPAI-positive cases, demonstrating that these findings can serve as a valuable reference for rapid disease control management. While genetic analysis or histological experimental results were not included, this study provides valuable insights based on macroscopic observations attributed to natural infection with HPAI.

## Data Availability

The data generated or analyzed during this study are available from the corresponding author upon reasonable request.

## Nomenclature

HPAI: High pathogenicity avian influenza
AIV: Avian influenza virus
HPAIV: High pathogenicity avian influenza virus
HA: Hemagglutinin
ADC: Animal Disease Control
APQA: Animal and Plant Quarantine Agency.

## Supporting Information

Supporting Information 1 Numbers of poultry farms infected with HPAI virus belonged to the H5 subtype clade 2.3.4.4b in South Korea between 2023 and 2025.

Supporting Information 2 Summary the individual HPAI outbreaks analyzed in this study.
