# Supplementary material for: Comparison of Gross Lesions in Poultry Naturally Infected With High Pathogenicity Avian Influenza H5N6 and H5N1 Viruses in South Korea, 2023–2025
Source: Transbound Emerg Dis. 2025 Nov 25;2025:1736453. doi: 10.1155/tbed/1736453 (PMC12672067; doi:10.1155/tbed/1736453)
Supplement: Supporting Information 1 — Numbers of poultry farms infected with HPAI virus belonged to the H5 subtype clade 2.3.4.4b in South Korea between 2023 and 2025. [file 1736453.f1.docx]

**Supplementary Table 1.** Numbers of poultry farms infected with HPAI virus belonged to the H5 subtype clade 2.3.4.4b in South Krea between 2023 and 2025.

| **Species** | **Farms** | **2023 season** | **2024-2025 season** |
| --- | --- | --- | --- |
| Chicken | Layer | 10 | 17 (2) |
|  | Broiler-breeder | 2 | 2 |
|  | Layer-breeder | - | 1 |
|  | Korean native chicken | - | 2 |
| Duck | Breeder duck | 1 | 2 (1) |
|  | Meat duck | 3 (1) | 9 (4) |
| Total |  | 16 | 33 |

* The number in parentheses is the number of farms diagnosed with HPAI through active surveillance.
